# The association between angiographic stenosis characteristics and surgical treatment in diffuse LAD disease

**DOI:** 10.1016/j.ijcha.2026.102000

**Published:** 2026-08-25

**Authors:** Matteo Betti, Roberto Bova, Samuel Heuts, Pieter A. Vriesendorp, Alexander J.J. Ijsselmuiden, Saman Rasoul, Mustafa Ilhan, Jindra Vainer, Ralph A.L.J. Theunissen, Leo F. Veenstra, Anass Maaroufi, Daniek P.J. Slegers, Arnoud W.J. van 't Hof, Arpad Lux

**Affiliations:** a Department of Cardiology, Maastricht University Medical Centre+, Maastricht, the Netherlands; b Cardiovascular Research Institute Maastricht, Maastricht, the Netherlands; c Department of Cardiothoracic surgery, Maastricht University Medical Centre+, Maastricht, the Netherlands; d Department of Cardiology, Zuyderland Medical Center, Heerlen, the Netherlands

**Keywords:** Coronary physiology, Pullback, Diffuse disease, Revascularization, CABG

## Abstract

**Background:**

Intracoronary pressure-wire pullback allows differentiation between focal and diffuse disease, improving precision of revascularisation strategies. While focal lesions benefit from percutaneous coronary intervention (PCI), diffuse disease yields suboptimal results and is therefore treated with coronary artery bypass grafting (CABG) or conservatively (i.e., optimal medical therapy [OMT]). Current guidelines emphasise the prognostic relevance of proximal left anterior descending artery (LAD) disease, yet its role in guiding treatment in diffuse disease remains uncertain.

**Objectives:**

To evaluate whether angiographically severe proximal LAD disease influences revascularisation strategy in functionally significant diffuse LAD disease.

**Methods:**

This retrospective cohort study included patients undergoing coronary angiography and subsequent pullback measurement in the LAD in the presence of ischemia (FFR ≤0.80 or RFR ≤0.89), between 2022 and 2024. Coronary anatomy was reassessed blinded to treatment. Multinomial logistic regression explored predictors of treatment strategy (OMT, PCI, or CABG) in patients with diffuse LAD disease.

**Results:**

Among 303 patients with significant LAD disease, 214 (71%) had diffuse and 89 (29%) focal disease. Baseline characteristics were similar. Focal disease was more associated with triple-vessel CAD (34% vs. 19%; *p* = 0.007). Severe proximal (21% vs. 7.9%; *p* = 0.004) and distal LAD stenosis were more common in diffuse disease. In diffuse disease only, severe proximal LAD was associated with an increase in CABG (28.3% vs. 10.1%; *p* = 0.004) and a decrease in OMT rates (54.3% vs. 75.6%; *p* = 0.009). Multinomial regression confirmed this association (OR 0.26; *p* = 0.003).

**Conclusion:**

Absence of angiographically severe proximal LAD stenosis is associated with CABG deferral in diffuse, functionally significant LAD disease.

## 1 Introduction

Contemporary percutaneous coronary revascularization strategy has shifted from angiography to physiology-informed decision-making, with pressure-derived indices (Fractional Flow Reserve [FFR] and non-hyperaemic ratios) improving patient selection and outcomes across chronic and acute coronary syndromes [1], [2], [3], [4], [5]. Once ischaemia is confirmed, the spatial distribution of pressure loss along the vessel can be evaluated to anticipate the benefit of percutaneous coronary intervention (PCI) [6].

Intracoronary pressure-wire pullback has emerged as the reference method to phenotype disease as focal or diffuse based on the pattern of haemodynamic impairment [7]. Pullback findings are often discordant with angiography, particularly in diffuse disease where visual severity may be at odds with functional severity [8]. While focal lesions derive clear benefit from PCI due to localised flow-limiting obstruction, stenting of diffuse disease yields less hemodynamic improvement and is more frequently followed by residual angina and worse clinical outcomes compared with treatment of focal disease [6], [9], [10]. Consequently, diffuse coronary artery disease (CAD) is often referred for surgical revascularization or managed conservatively (i.e. optimal medical therapy [OMT]).

Our working group recently underscored the strong influence of physiological lesion characteristics, including lower hyperaemic thresholds, on treatment selection, demonstrating that diffuse disease not only reduces PCI rates but also leads to the withholding of coronary artery bypass grafting (CABG) [11]. We hypothesised that the prognostic relevance of significantly decreased FFR values in the setting of diffuse disease could be overruled by angiographic vessel characteristics – specifically, the absence of obvious flow-limiting lesions – leading to the deferral or rejection of CABG. This decision may be driven by a perceived risk of graft failure or concerns regarding the clinical meaningfulness of the procedure itself [12], [13]. Therefore, although pressure-wire pullback can reclassify disease patterns and change treatment strategy, we hypothesised that coronary anatomy may still carry substantial weight in surgical decision-making beyond pullback findings [8]. To address this gap, we investigated whether severe proximal stenosis influences referral for CABG in patients with diffuse, functionally significant left anterior descending artery (LAD) disease.

## 2 Methods

### 2.1 Study design and population

This retrospective cohort study was approved by the local Medical Ethical Committee (METC) on 04/10/2022 (number 2022–3303) and was conducted at Maastricht University Medical Center+ (Maastricht, the Netherlands). We enrolled adults (≥18 years) presenting with stable angina, unstable angina, or non-ST-elevation myocardial infarction (NSTEMI) who underwent coronary angiography and had physiologically significant LAD disease—defined as FFR ≤0.80 or resting full-cycle ratio (RFR) ≤0.89—between January 1, 2022, and January 1, 2024. Patients were excluded for a STEMI within 30 days before angiography, in-stent restenosis, previous CABG, and presence of left main coronary artery disease. For each patient, the treatment performed after Heart Team discussion was recorded.

### 2.2 Data collection

Clinical information and baseline characteristics were obtained from electronic patient records, prospectively collected Heart Team documentation, and coronary angiography reports and images. All coronary angiograms were reviewed in IntelliSpace Cardiovascular (Philips, Amsterdam, the Netherlands) by an interventional cardiologist; the LAD was assessed per-segment (proximal, mid, distal) for visual diameter stenosis (DS) severity (graded as mild [30–50% DS], moderate [50–70% DS] and severe [≥70% DS]) and visible calcification (recorded as present or absent). Patients were then classified according to the presence or absence of severe proximal LAD disease (i.e., the segment from the ostium to the first major septal or diagonal). Clinical follow-up data at 1 year were available for all included patients, and no patients were lost to follow-up.

### 2.3 Intracoronary measurements

FFR and RFR were obtained with the PressureWire™ X guidewire interfaced with the CoroFlow‡ Cardiovascular System (Abbott Cardiovascular). In the majority of cases, both FFR and RFR were measured sequentially during the same procedure, while in a small proportion of patients (4.3%) only RFR was available. RFR pullback was systematically performed in all patients during manual pullback at the time of coronary angiography to provide a continuous pressure profile along the vessel. Pullback phenotype classification (focal vs diffuse) was performed prospectively by the first operator during the index procedure and documented in the procedural report; no retrospective adjudication was performed for the purpose of this analysis. Functional focal disease was defined by an abrupt pressure step-up (>75% of the total pressure drop) at one or two points along the coronary artery, whereas diffuse disease was defined by a gradual pressure increase throughout the vessel, according to previously published pressure-wire pullback criteria [11], [14]. These definitions are also being used in the ongoing prospective multicentre COMMIT-LAD registry [15]. For the present analysis, only functionally significant LAD lesions were included (FFR ≤0.80 or RFR ≤0.89).

### 2.4 Statistical analysis

Continuous variables were reported as means with standard deviations or as medians with interquartile ranges, depending on their distribution, which was tested with Shapiro-Wilk test. Categorical variables were presented as absolute counts and corresponding percentages.

Comparative analyses between groups were conducted using appropriate statistical tests. Continuous variables were analysed using either ANOVA or the Kruskal-Wallis test, depending on the data distribution. Categorical variables were compared using the Chi-square test or Fisher's exact test (in case of cell count <5) as appropriate.

To address missing data, a pairwise deletion approach was applied: analyses were conducted using all available data for each specific comparison, while missing values in unrelated variables were excluded.

The primary outcome was to evaluate whether visually severe proximal LAD involvement influences CABG referral in patients with functionally significant diffuse LAD disease. To adjust for potential confounders, a multinomial regression analysis was performed to evaluate the association between baseline variables, coronary characteristics, and eventual treatment strategy. Variables were selected a priori based on their established clinical relevance and previously reported association with treatment decision and long-term outcomes after CABG [16]. To reduce the risk of model overfitting, given the limited number of patients undergoing CABG, only a limited number of clinically relevant covariates were included in the final model. The eventual effect was expressed in an odds ratio (OR) with a 95% confidence interval (CI) for PCI or OMT, relative to CABG (i.e., CABG as the reference category).

The composite endpoint of major adverse cardiovascular events (MACE) was defined as the occurrence of all-cause death, myocardial infarction, urgent revascularization, or stroke within 1 year. Clinical outcomes were compared across treatment groups (i.e., OMT, PCI, and CABG) in patients showing diffuse disease at pullback using the Chi-square or Fisher's exact test, as appropriate, depending on cell counts.

All statistical tests were two-sided, and *P* values <0.05 were considered statistically significant. All statistical analyses were performed using R (version 4.4.2, R Foundation for Statistical Computing, Vienna, Austria).

## 3 Results

### 3.1 Patient demographics and characteristics

**Fig. 1:**
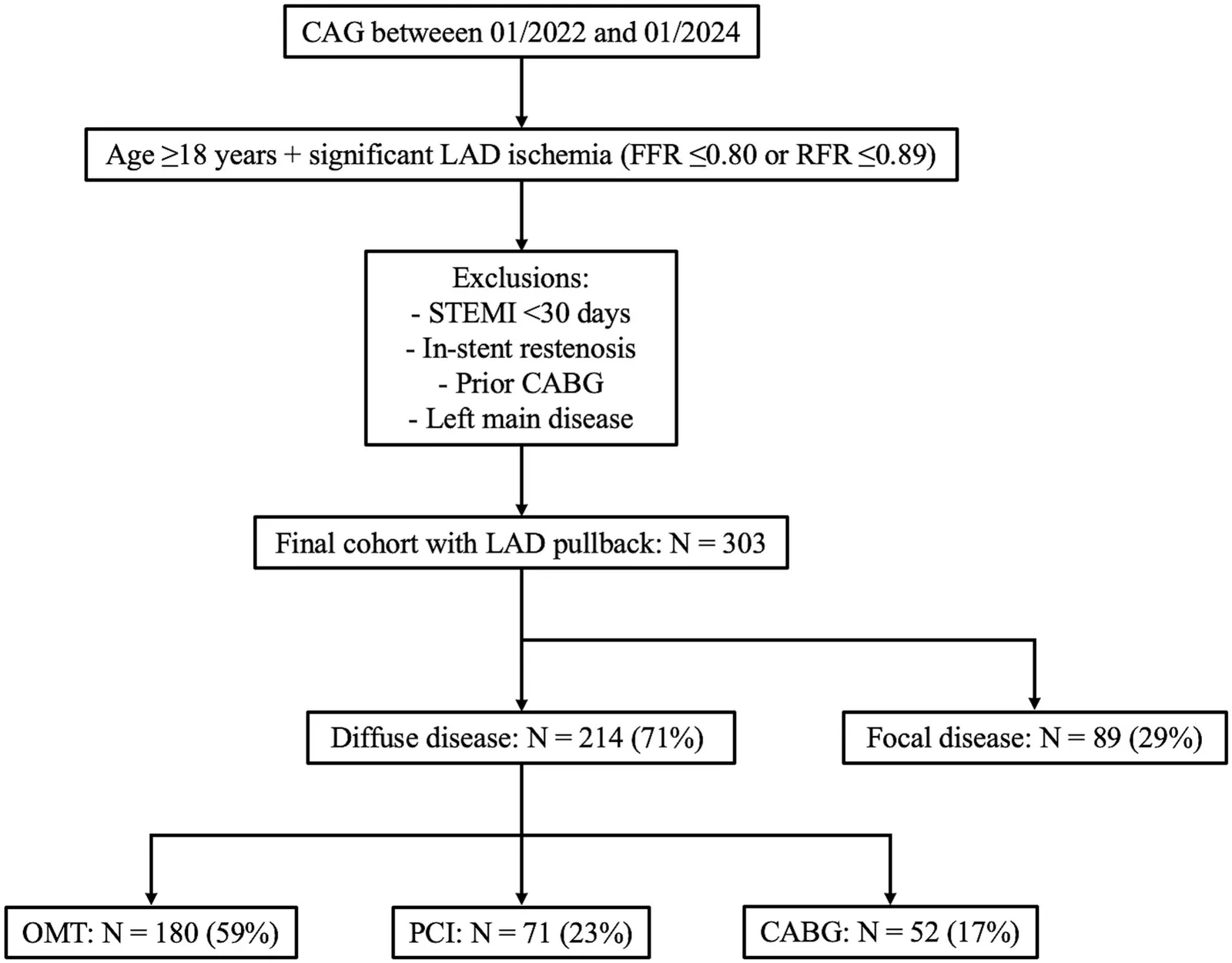
Flowchart of patient selection. Patients with physiologically significant LAD disease (FFR ≤0.80 or RFR ≤0.89) undergoing pressure-wire pullback were classified as having diffuse or focal disease according to the pullback pattern and included in the subsequent analyses. CABG = coronary artery bypass grafting; CAG = coronary angiogram; FFR = Fractional Flow Reserve; LAD = left anterior descending; OMT = optimal medical therapy; PCI = percutaneous coronary intervention; RFR = Resting Full-Cycle Ratio; STEMI = ST-elevation myocardial infarction.

The present study included 303 patients with functionally significant LAD disease, who received RFR pullback measurement to distinguish its disease pattern. 214 patients (71%) had diffuse disease, whereas 89 (29%) had focal disease (Fig. 1).

Baseline characteristics across pullback patterns are summarised in Table 1. Patients with diffuse and focal disease had broadly comparable demographic, clinical, and laboratory characteristics. The only significant differences were a higher prevalence of atrial fibrillation (23% vs. 9%; *p* = 0.004) and a lower prevalence of three-vessel disease (19% vs. 34%; *p* = 0.007) in patients with diffuse disease. FFR values were similar between groups, whereas median RFR was slightly higher in diffuse disease (*p* = 0.022).

**Table 1:** Baseline patient characteristics across CAD phenotypes. Symptom score was defined as 0 = no symptoms, 1 = either dyspnoea or angina, 2 = both symptoms.

	Diffuse disease (*N* = 214)	Focal disease (*N* = 89)	Overall (*N* = 303)	*p*-value
Demographics				
Age (years)	68 [62–75]	69 [62–76]	68 [62–75]	0.800
Sex (male)	170 (79%)	66 (74%)	236 (78%)	0.362
BMI (kg/m2)	27.26 [24.22–30.04]	26.37 [24.33–28.91]	26.96 [24.22–29.60]	0.173

Risk factors				
Diabetes mellitus	43 (20%)	16 (18%)	59 (19%)	0.751
Hypertension	148 (69%)	66 (74%)	214 (71%)	0.409
Dyslipidaemia	109 (51%)	54 (61%)	163 (54%)	0.131

Smoking status				0.361
Never smoked	102 (48%)	36 (40%)	138 (46%)	
History of smoking	81 (38%)	35 (39%)	116 (38%)	
Active smoker	31 (14%)	18 (20%)	49 (16%)	

Comorbidities				
COPD	22 (10%)	5 (5.6%)	27 (8.9%)	0.268
CVA	26 (12%)	9 (10%)	35 (12%)	0.696
PAD	13 (6.1%)	6 (6.7%)	19 (6.3%)	0.799
PH	18 (8.4%)	7 (7.9%)	25 (8.3%)	>0.999
Atrial fibrillation	50 (23%)	8 (9.0%)	58 (19%)	**0.004**
Prior myocardial infarction	49 (23%)	22 (25%)	71 (23%)	0.767
Prior stable CAD	97 (45%)	37 (42%)	134 (44%)	0.612

Symptoms				
Dyspnoea	139 (65%)	63 (71%)	202 (67%)	0.352
Angina pectoris	166 (78%)	66 (74%)	232 (77%)	0.553
Symptom score				0.782
0	22 (10%)	10 (11%)	32 (11%)	
1	79 (37%)	29 (33%)	108 (36%)	
2	113 (53%)	50 (56%)	163 (54%)	

Measurements				
LVEF	56 [49–61]	56 [50–60]	56 [49–61]	0.969
EuroSCORE II	1.06 [0.73–1.80]	1.06 [0.76–1.78]	1.06 [0.74–1.79]	0.890
Creatinine	88 [76–100]	87 [72–101]	87 [75–100]	0.949
eGFR	70.80 [59.80–81.00]	73.00 [57.55–82.60]	71.10 [59.80–81.40]	0.643
LDL-cholesterol	2.30 [1.80–3.20]	2.40 [1.90–3.80]	2.30 [1.80–3.25]	0.197
HDL-cholesterol	1.20 [1.00–1.50]	1.20 [1.00–1.50]	1.20 [1.00–1.50]	0.983
Total cholesterol	4.30 [3.60–5.30]	4.40 [3.70–5.60]	4.30 [3.60–5.40]	0.314
Haemoglobin	8.90 [8.10–9.50]	8.70 [8.20–9.50]	8.85 [8.10–9.50]	0.955

CAG findings				
Number of vessel disease				**0.017**
1	106 (50%)	33 (37%)	139 (46%)	0.058
2	68 (32%)	26 (29%)	94 (31%)	0.685
3	40 (19%)	30 (34%)	70 (23%)	**0.007**
FFR	0.76 [0.72–0.78]	0.74 [0.68–0.78]	0.75 [0.71–0.78]	0.101
RFR	0.86 [0.81–0.88]	0.84 [0.75–0.88]	0.86 [0.79–0.88]	**0.022**

BMI = body mass index; CAD = coronary artery disease; CAG = coronary angiography; COPD = chronic obstructive pulmonary disease; CVA = cerebrovascular accident; eGFR = estimated glomerular filtration rate; FFR = Fractional Flow Reserve; LVEF = left ventricular ejection fraction; PAD = peripheral artery disease; PH = pulmonary hypertension; RFR = Resting Full-Cycle Ratio.

### 3.2 Angiographic LAD characteristics and pullback phenotype

LAD anatomical characteristics across patients with diffuse and focal disease are shown in Table 2. Assessment of angiographic LAD anatomy revealed more proximal and distal severe stenosis in patients with diffuse disease compared with those with focal disease. Specifically, severe proximal LAD stenosis was significantly more frequent in diffuse disease (21% vs. 7.9%; *p* = 0.004), as was distal LAD ≥70% DS (8.9% vs. 1.1%; *p* = 0.010). In contrast, no significant differences were observed in the prevalence of moderate stenosis (50–70% DS) or calcifications across the proximal, mid, or distal LAD segments.

**Table 2:** Angiographic LAD characteristics and pullback phenotype.

	Diffuse disease (N = 214)	Focal disease (N = 89)	Overall (N = 303)	p-value
Proximal LAD calcification	73 (34%)	28 (31%)	101 (33%)	0.690
Proximal LAD 50–70% DS	80 (37%)	27 (30%)	107 (35%)	0.291
Proximal LAD ≥70% DS	46 (21%)	7 (7.9%)	53 (17%)	**0.004**
Mid LAD calcification	59 (28%)	29 (33%)	88 (29%)	0.406
Mid LAD 50–70% DS	119 (56%)	45 (51%)	164 (54%)	0.449
Mid LAD ≥70% DS	49 (23%)	29 (33%)	78 (26%)	0.085
Distal LAD calcification	8 (3.7%)	1 (1.1%)	9 (3.0%)	0.291
Distal LAD 50–70% DS	44 (21%)	11 (12%)	55 (18%)	0.103
Distal LAD ≥70% DS	19 (8.9%)	1 (1.1%)	20 (6.6%)	**0.010**

DS = diameter stenosis; LAD = left anterior descending artery.

### 3.3 Association between severe proximal LAD disease and treatment decision

**Fig. 2:**
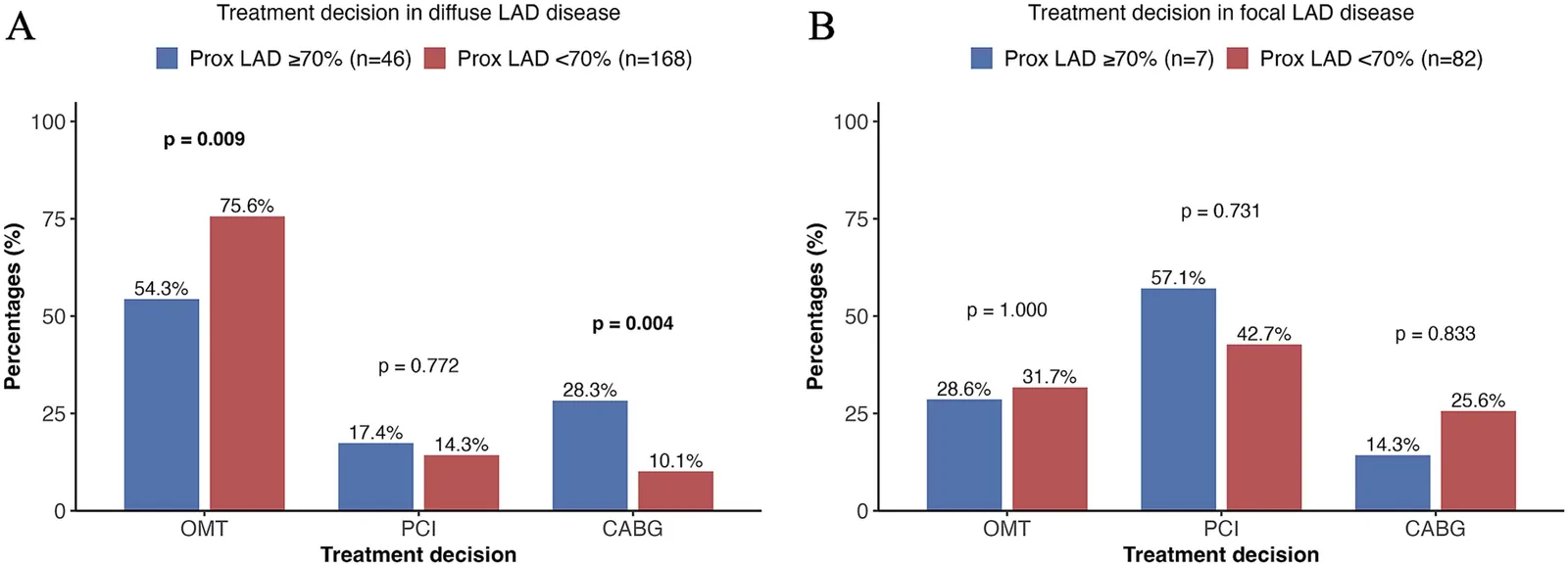
Treatment strategy according to the presence or absence of angiographically severe proximal LAD stenosis (≥70% diameter stenosis), stratified by pullback phenotype. Panel A shows patients with diffuse disease and Panel B patients with focal disease. Bars represent the proportion of patients treated with OMT, PCI, or CABG. *P* values refer to comparisons between patients with and without severe proximal LAD stenosis within each pullback phenotype. CABG = coronary artery bypass grafting; LAD = left anterior descending artery; OMT = optimal medical therapy; PCI = percutaneous coronary intervention.

The association between severe proximal LAD disease and treatment strategy differed according to pullback phenotype. In diffuse disease, severe proximal LAD was associated with a higher proportion of patients undergoing CABG (28.3% vs. 10.1%; *p* = 0.004) and a lower proportion receiving OMT (54.3% vs. 75.6%; *p* = 0.009), while PCI use was similar (17.4% vs. 14.3%; *p* = 0.772) (Fig. 2A). In focal disease, treatment distribution did not differ significantly by proximal LAD severity (OMT 28.6% vs 31.7%, PCI 57.1% vs 42.7%, CABG 14.3% vs 25.6%; all *p* ≥ 0.73) (Fig. 2B).

### 3.4 Predictors of revascularization strategy

To assess the influence of severe proximal LAD disease among other factors on treatment decision in patients with diffuse LAD disease, multinomial logistic regression analyses were conducted (Table 3). In our multinomial regression using CABG as the reference category, all clinical predictors including age, sex, diabetes, chronic obstructive pulmonary disease, prior cerebrovascular events, and left ventricular ejection fraction were not significantly associated with the likelihood of receiving PCI or OMT compared with CABG. In contrast, severe proximal LAD stenosis was independently associated with a lower likelihood of being treated with OMT rather than CABG (OR 0.26; 95% CI 0.11–0.65; *p* = 0.003), while no significant association was found for PCI versus CABG. Likewise, no significant associations were observed between any predictors and the selection of PCI over OMT.

**Table 3:** Multivariable logistic regression analysis showing predictors of treatment strategy in patients with diffuse LAD disease.

	OMT relative to CABG			PCI relative to CABG			PCI relative to OMT		
	OR	95% CI	p-value	OR	95% CI	p-value	OR	95% CI	p-value
Sex (male)	0.37	0.10–1.39	0.140	0.32	0.07–1.48	0.144	0.87	0.33–2.31	0.782
Age (years)	1.01	0.96–1.06	0.670	1.01	0.94–1.07	0.861	0.99	0.95–1.04	0.831
3VD	0.73	0.24–2.20	0.571	1.46	0.39–5.39	0.573	2.01	0.74–5.42	0.169
DM	0.88	0.28–2.75	0.821	1.01	0.26–3.94	0.987	1.15	0.42–3.15	0.780
COPD	1.02	0.24–4.34	0.980	0.87	0.14–5.27	0.881	0.86	0.21–3.44	0.826
CVA	3.42	0.42–27.92	0.250	3.88	0.39–38.58	0.247	1.13	0.34–3.78	0.837
LVEF	0.98	0.94–1.02	0.367	0.98	0.93–1.03	0.467	1.00	0.96–1.04	0.978
Proximal LAD ≥70% DS	0.26	0.11–0.65	**0.003**	0.43	0.14–1.35	0.149	1.63	0.61–4.37	0.332

3VD = three-vessel disease; CABG = coronary artery bypass grafting; COPD = chronic obstructive pulmonary disease; CVA = cerebrovascular accident; DM = diabetes mellitus; DS = diameter stenosis; LAD = left anterior descending artery; LVEF = left ventricular ejection fraction; PCI = percutaneous coronary intervention; OMT = optimal medical therapy.

### 3.5 Clinical outcomes

At one year, clinical event rates were low across treatment strategies. The incidence of MACE was 9.9% in the OMT group, 9.4% in the PCI group, and 6.7% in the CABG group (*p* = 0.934), with no significant differences observed for all-cause death, myocardial infarction, urgent revascularization, or stroke (all *p* > 0.45) (Table 4). Kaplan-Meier analysis likewise demonstrated no significant difference in the cumulative incidence of MACE among treatment strategies (log-rank *p* = 0.874; Supplementary Material S1).

**Table 4:** 1-year clinical outcomes in patients with diffuse LAD disease. MACE was defined as a composite of all-cause death, myocardial infarction, urgent revascularization, or stroke.

	OMT (*N* = 152)	PCI (*N* = 32)	CABG (*N* = 30)	Overall (*N* = 214)	p-value
MACE	15 (9.9%)	3 (9.4%)	2 (6.7%)	20 (9.3%)	0.934
All-cause death	7 (4.6%)	2 (6.3%)	0 (0%)	9 (4.2%)	0.488
Myocardial infarction	4 (2.6%)	1 (3.1%)	1 (3.3%)	6 (2.8%)	1.000
Urgent revascularization	3 (2.0%)	1 (3.1%)	1 (3.3%)	5 (2.3%)	0.451
Stroke	4 (2.6%)	0 (0%)	1 (3.3%)	5 (2.3%)	0.808

CABG = coronary artery bypass grafting; MACE = major adverse cardiovascular events; OMT = optimal medical therapy; PCI = percutaneous coronary intervention.

## 4 Discussion

The present study evaluated whether angiographic stenosis characteristics influence treatment allocation beyond physiological assessment in patients with diffuse LAD disease. Severe proximal LAD disease was associated with a higher likelihood of referral for CABG and a lower likelihood of pharmacological management, whereas its absence was associated with deferral of surgery despite objective evidence of diffuse ischaemia. These findings provide novel evidence that coronary anatomy influences surgical decision making beyond pullback findings.

The management of CAD has improved ever since pullback measurements were introduced as they allow a precise distinction of the atherosclerosis phenotypes, focal and diffuse disease, therefore improving revascularization outcomes [8]. It is established that PCI can improve intracoronary hemodynamics, patient's angina status and clinical outcomes in the setting of focal CAD [10]. On the contrary, PCI yields suboptimal results in diffuse disease, and these patients are usually referred to CABG. Notwithstanding, our guidelines do not give advice on how to address diffuse single-vessel disease and only recommend surgical revascularization when there is involvement of the proximal LAD [12].

The efficacy of a pullback-guided PCI strategy has been demonstrated across the spectrum of both chronic and acute coronary syndromes, exception made for STEMI [17]. A recent work from our group already demonstrated that RFR pullback curves significantly affect treatment decision in this scenario [18]. In addition, a prospective study from our institution (COMMIT-LAD) is currently enrolling patients with significant LAD disease to investigate whether a standardized physiological assessment can improve clinical outcomes, with 1-year MACE and patient-reported outcomes as primary endpoints [15], [19]. The present study builds on these findings by investigating whether coronary anatomy continues to influence treatment allocation after physiological assessment has identified diffuse LAD disease. Accordingly, the study population comprised patients undergoing coronary angiography with subsequent manual pressure-wire RFR pullback for stable angina, unstable angina, or NSTEMI. All pullback measurements were performed at the time of coronary angiography, at the discretion of the operator, and were not demanded by the Heart Team.

More than two-thirds (71%) of our patients showed diffuse disease, whereas only 29% focal disease. This distribution is consistent with recent evidence showing that physiologically diffuse disease is at least as common and often more frequent than focal disease [7], [20]. Patients with diffuse and focal LAD disease exhibited comparable demographic and clinical characteristics, supporting the concept that atherosclerosis phenotypes are not readily explained by differences in traditional cardiovascular risk factors or comorbidity burden. There were, however, angiographic differences: in patients with diffuse disease, we observed a broader distribution of severe stenosis involving both proximal and distal LAD segments as compared to focal disease. Interestingly, in this cohort, focal disease was more common in patients with triple-vessel disease. Despite these angiographic differences, FFR was similar between groups, while diffuse disease showed slightly higher median RFR values than focal disease. Although the absolute difference was small and unlikely to be clinically relevant, it is consistent with the distinct physiological pattern of diffuse coronary artery disease, characterised by gradual pressure loss along the vessel [21], [22].

In patients with diffuse disease, angiographically severe (≥70% DS) proximal LAD stenosis was associated with a shift from medical therapy towards surgical revascularization, a pattern that was not observed in patients with focal disease. All included patients had confirmed ischemia. Therefore, they could have benefited from surgical revascularization of territories affected by significant stenoses through a reduction of ischemic burden and prevention of future myocardial infarctions [23], [24]. In patients with diffuse disease, the lack of visible flow-limiting disease in the proximal LAD might raise concerns for graft failure due to a competitive flow and result in rejection of surgical revascularisation [25], [26]. Moreover, this reflects an established practice, which is to weight anatomical complexity in decision-making for surgery: patients referred to CABG had not only more frequently multivessel disease, but also severe calcifications in the proximal LAD, potentially increasing the SYNTAX score (Supplementary Material S2). On the other hand, diffuse disease and long lesions are also elements that increase the SYNTAX score, but despite this, revascularization is often not the first option for these patients [27].

To further understand the association of severe proximal LAD disease and referral to CABG in diffuse disease, we conducted a multinomial regression analysis adjusting for other important clinical predictors that have a weight on treatment decision [16]. Using CABG as the reference category, severe proximal LAD disease was independently associated with a lower likelihood of OMT. This underscores the role of visible proximal LAD involvement in favouring surgical revascularization, beyond the presence of significant ischaemia and diffuse pressure loss identified by pullback assessment.

As expected, patients with diffuse disease on pullback assessment were more frequently managed conservatively, with a predominance of OMT (71%) [11]. Despite this imbalance in treatment allocation, 1-year clinical outcomes were similar across strategies, with no differences observed in MACE and its individual components. These findings are consistent with prior evidence indicating that, in both stable coronary artery disease and non-ST-segment-elevation acute coronary syndromes, an initial conservative strategy may represent a safe and reasonable option in selected patients [27], [28], [29], [30]. However, the study was not powered to detect differences in clinical endpoints. Given the limited number of patients in the PCI and CABG groups and the low number of MACE events, these analyses should be considered exploratory and hypothesis-generating. Therefore, no conclusion regarding the comparative prognostic efficacy or safety of the three treatment strategies can be drawn from the present data.

While the prognostic benefit of invasive treatment for proximal LAD disease is already known, uncertainty remains regarding the best treatment option for diffuse LAD disease. These patients may represent a higher-risk population, and the long-term outcomes regarding revascularization versus conservative therapy in this context are still unknown. Further evidence is needed to confirm the safety of a conservative strategy in the absence of angiographic “prognostically significant” lesions in diffuse functionally significant LAD disease. These findings provide new insight into how anatomical and physiological information are integrated in contemporary clinical practice and identify an important evidence gap that should be addressed in future prospective studies.

### 4.1 Limitations of the study

First, this was a retrospective observational study conducted at a single centre, which may limit the generalisability of our findings. Second, anatomical vessel data were not standardized and did not include factors relevant to CABG indication (e.g., distal reference vessel diameter, bifurcation lesions, SYNTAX score), potentially introducing residual confounding. Third, pullback phenotypes were classified prospectively by the treating operators without independent core laboratory adjudication. Although this approach reflects routine clinical practice and avoids retrospective review bias, some degree of interobserver variability and subjective interpretation cannot be excluded. Fourth, the present findings have not been externally validated and should therefore be interpreted with caution until confirmed in independent cohorts. Finally, follow-up was limited to one year, precluding conclusions regarding the long-term comparative efficacy and safety of the different treatment strategies.

## 5 Conclusions

In patients with diffuse, functionally significant LAD disease, severe proximal LAD stenosis is associated with a greater likelihood of referral for CABG, whereas its absence is associated with surgical deferral despite physiological evidence of diffuse ischaemia. Further studies are needed to establish the optimal treatment strategy and its impact on long-term clinical outcomes in this patient population.

## CRediT authorship contribution statement

**Matteo Betti:** Writing – original draft, Visualization, Software, Resources, Methodology, Investigation, Data curation, Conceptualization. **Roberto Bova:** Resources, Project administration, Methodology, Investigation, Data curation, Conceptualization. **Samuel Heuts:** Writing – review & editing, Supervision, Methodology. **Pieter A. Vriesendorp:** Investigation. **Alexander J.J. Ijsselmuiden:** Investigation. **Saman Rasoul:** Investigation. **Mustafa Ilhan:** Investigation. **Jindra Vainer:** Investigation. **Ralph A.L.J. Theunissen:** Investigation. **Leo F. Veenstra:** Investigation. **Anass Maaroufi:** Investigation. **Daniek P.J. Slegers:** Investigation, Data curation. **Arnoud W.J. van 't Hof:** Writing – review & editing, Supervision, Investigation. **Arpad Lux:** Writing – review & editing, Supervision, Methodology, Investigation.

## Funding

No funding involved.

## Declaration of competing interest

S.R. received research grants from Abbott Vascular, consulting fees from Philips, and advisory fees from Amgen and Novartis. A.H. received research grants from Medtronic, Astra Zeneca, Boehringer-Ingelheim and consulting fees from Celecor Therapeutics. A.L. received research grants from Abbott Vascular and speakers' honoraria from Johnson & Johnson Medical.
